# A Combined Gravity Compensation Method for INS Using the Simplified Gravity Model and Gravity Database

**DOI:** 10.3390/s18051552

**Published:** 2018-05-14

**Authors:** Xiao Zhou, Gongliu Yang, Jing Wang, Zeyang Wen

**Affiliations:** 1 School of Instrument Science and Opto-Electronics Engineering, Beihang University, Beijing 100191, China; yanggongliu@buaa.edu.cn (G.Y.); by1717133@buaa.edu.cn (Z.W.); 2 Science and Technology on Inertial Laboratory, Beihang University, Beijing 100191, China; 3 School of Mechatronices and Information Engineering, China Mining University, Beijing 100083, China; by1217133@buaa.edu.cn

**Keywords:** error modelling, gravity model, extreme learning machine (ELM), gravity compensation, high precision free-INS

## Abstract

In recent decades, gravity compensation has become an important way to reduce the position error of an inertial navigation system (INS), especially for a high-precision INS, because of the extensive application of high precision inertial sensors (accelerometers and gyros). This paper first deducts the INS’s solution error considering gravity disturbance and simulates the results. Meanwhile, this paper proposes a combined gravity compensation method using a simplified gravity model and gravity database. This new combined method consists of two steps all together. Step 1 subtracts the normal gravity using a simplified gravity model. Step 2 first obtains the gravity disturbance on the trajectory of the carrier with the help of ELM training based on the measured gravity data (provided by Institute of Geodesy and Geophysics; Chinese Academy of sciences), and then compensates it into the error equations of the INS, considering the gravity disturbance, to further improve the navigation accuracy. The effectiveness and feasibility of this new gravity compensation method for the INS are verified through vehicle tests in two different regions; one is in flat terrain with mild gravity variation and the other is in complex terrain with fierce gravity variation. During 2 h vehicle tests, the positioning accuracy of two tests can improve by 20% and 38% respectively, after the gravity is compensated by the proposed method.

## 1. Introduction

Achieving accurate navigation is crucial for the modern motion carrier. Nowadays the most common way to navigate for most modern vehicles and aircrafts is utilizing the global positioning system (GPS). The significant advantage of GPS is its real-time high precision positioning with the help of satellites’ signals. However, GPS cannot receive the satellites’ signals in some circumstances due to the physical blockages, such as in a tunnel or underwater. The inertial navigation system (INS) utilizes Newton’s law to realize autonomous navigation worldwide, and the initialization errors propagate throughout the trajectory with the increase in time. Although the long-term navigation accuracy of free-INS cannot compare with that of GPS, it is still essential during times of loss of GPS. Because INS can work in all weather conditions, has high anti-jamming capacity, and radiates no signal to the outside, INS has been used for many military and civil applications [1]. In recent years, with the emergence of cold atom interferometry, developing high precision INS has become possible. It is predicted that the accelerometer and gyro of this INS can have an accuracy of 10^−8^ m/s^2^ and 10^−6^ deg/h, respectively, which is at least 100 times better than the current INS. The significant improvement of INS, especially on inertial sensors, leaves the gravity compensation as the most important part effecting the positioning accuracy of INS, particularly for rough topological areas [2].

Normally, there are mainly three viable means to realize gravity compensation for INS [3]. Firstly there is the conventional method that uses gravitational gradiometers to gain the gravity disturbance on the trajectory [4,5]. This case is hard to apply to engineering applications because of the high cost of devices. The second method is using gravity models (like WGS84, DQM2000, EGM2008, etc.) to calculate the gravity disturbance and compensate it into the error equations of INS [6,7]. This method is easy to operate but the complexity of these models leads to a long time needed for a solution, which will hinder the real-time navigation of INS. The third method is to obtain the gravity disturbance with the help of the interpolation method based on a gravity database and compensate it into error equations of INS incorporated with gravity disturbance, which has a relatively ideal compensation result when gravity data is available [8,9,10,11,12]. Considering the characteristics of the second and the third method, in this paper, a combined gravity compensation method for INS using the simplified gravity model and gravity data is proposed to restrain the error propagation of INS. At last, two vehicle tests were applied to prove the effectiveness and precision of the proposed gravity compensation method.

The rest of this paper is organized as follows: Section 2 states the error analysis of INS solution, considering gravity disturbance. Section 3 presents the principle of the simplified gravity model. Section 4 introduces the theory and frame of the gravity data-based compensation method using ELM. Section 5 exhibits the framework of this combined gravity compensation method in INS. Section 6 performs vehicle tests in two different regions. Section 7 concludes the whole paper.

## 2. Error Analysis of INS Solution Caused by Gravity Disturbance

### 2.1. Definition of Gravity Disturbance Vector

**Figure 1:**
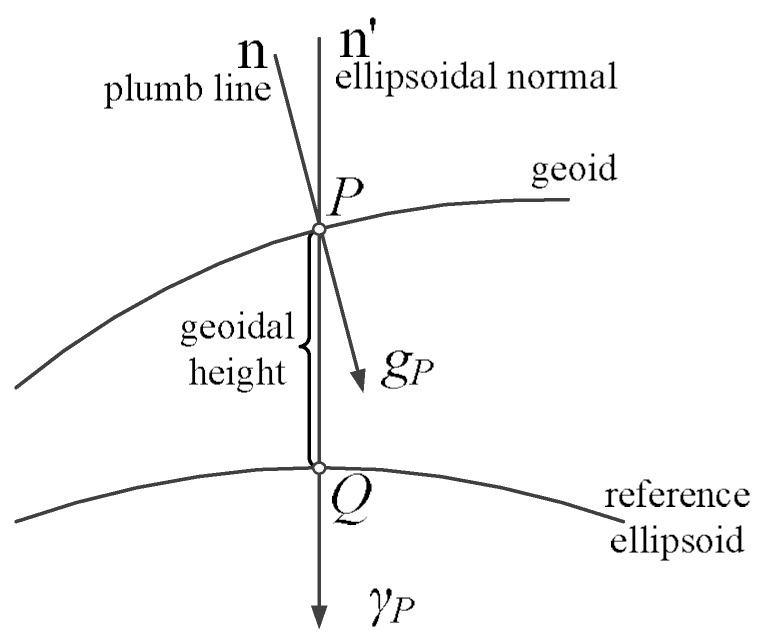
Description of gravity disturbance vector (GDV).

As Figure 1 shows, the gravity disturbance vector (GDV) is the vector difference between the normal gravity and the actual gravity on the same point in space. GDV is composed of two parts: the orthogonal component is denoted as gravity anomaly and the tangential component is denoted as vertical deflection [13].

In Figure 1, *g_p_* and *γ_p_* are defined as the gravity vector and the normal gravity vector at the point P, respectively. The difference between *g_p_* and *γ_p_* is defined as gravity disturbance vector *δg*:

(1) $$\delta g=g_{P}-\gamma_{P}$$

**Figure 2:**
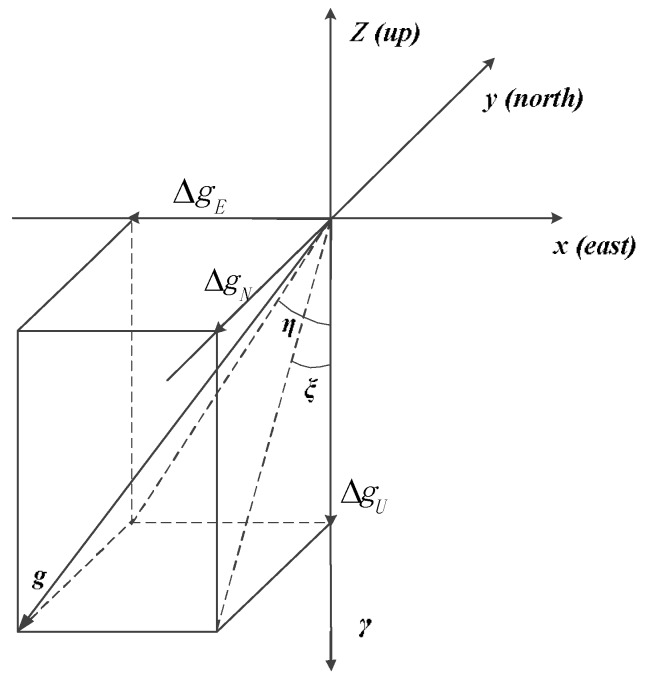
The deflection of the vertical (DOV).

As Figure 2 shows, the difference in direction is denoted as the deflections of the vertical (DOVs) [14,15]. The DOVs are composed of two components, *ζ* and *η* are represented as the north–south component and the east–west component, respectively.

(2) $$\delta g=[\Delta g_{E}\Delta g_{N}\Delta g_{U}]$$

(3) $$\left\{ \begin{array}{l} \delta g_{N}=-\gamma_{0}\zeta \\ \delta g_{E}=-\gamma_{0}\eta \end{array}\right.$$

where, Δg*_N_*, Δg*_E_*, and Δg*_U_* are presented as the north, east, and vertical components of gravity disturbance, respectively. *γ*_0_ is the value of normal gravity.

### 2.2. INS Error Equations Incorporated with Gravity Disturbance

The following INS error equations are considered with gravity disturbance [16,17,18,19]:

(4) $$\begin{array}{l} \delta {\dot{V}}^{n}=-\phi^{n}\times f^{n}+C_{b}^{n}(\delta K_{A}+\delta A)f^{b}+\delta V^{n}\times (2\omega_{ie}^{n}+\omega_{en}^{n})\\ +V\times (2\delta \omega_{ie}^{n}+\delta \omega_{en}^{n})+\nabla^{n}+\delta g^{n} \end{array}$$

(5) $$\left\{ \begin{array}{l} \delta \dot{L}=\frac{\delta V_{N}}{R_{M}+h}\\ \delta \dot{\lambda }=\frac{\delta V_{E}}{R_{N}+h}\sec L+\delta L\frac{V_{E}}{R_{N}+h}\tan L\sec L\\ \delta \dot{h}=\delta V_{U} \end{array}\right.$$

(6) $$\dot{\phi }=\phi \times \omega_{in}^{n}+\delta \omega_{in}^{n}-C_{b}^{n}(\delta K_{G}+\delta G)\omega_{ib}^{b}+\varepsilon^{n}$$

in the above formulas, $\delta V^{n}$ is defined as the velocity error in navigation frame, $\phi^{n}$ is defined as attitude error of the motion carrier, $f^{n}$ is defined as the specific force in navigation frame, $C_{b}^{n}$ is a direction cosine matrix which can be used to transform the body acceleration vector into the navigation frame, $\delta K_{A}$ and δA are defined as scale coefficient error and installation angle error of accelerometer respectively, δG and $\delta K_{G}$ are defined as installation angle error of gyros and scale coefficient error respectively, $\nabla^{n}$ is defined as the bias of accelerometers in navigation frame, $\delta g^{n}$ is defined as the gravity disturbance in navigation frame and $\varepsilon^{n}$ is the drift of gyros in navigation frame, $\delta \omega_{in}^{n}$ can be calculated from the following equation:

(7) $$\delta \omega_{in}^{n}=\delta \omega_{ie}^{n}+\delta \omega_{en}^{n}$$

where $\omega_{ie}^{n}$ and $\omega_{en}^{n}$ are presented as earth’s rotation rate and the navigation frame’s rotation with respect to earth respectively; both are expressed in the navigation frame. They can be calculated from the following formula:

(8) $$\delta \omega_{en}^{n}=\left[\begin{array}{c} -\frac{\delta V_{N}}{R_{M}+h}\\ \frac{\delta V_{E}}{R_{N}+h}\\ \frac{\delta V_{E}\tan L}{R_{N}+h}+\delta L\frac{V_{E}\sec^{2}L}{R_{N}+h} \end{array}\right]$$

(9) $$\delta \omega_{ie}^{n}={\left[0-\delta L\omega_{ie}\sin L\delta L\omega_{ie}\cos L\right]}^{T}$$

where L, λ, and h are denoted as the latitude, longitude, and altitude of the motion carrier respectively, $R_{M}$, $R_{N}$ are denoted as meridian radius and prime vertical radius, respectively.

From Equations (4)–(9), we could draw the conclusion that INS velocity errors $\delta V^{n}$ are mainly caused by the accelerometer’s output error $(\delta K_{A}+\delta A)f^{b}+\nabla^{n}$, velocity error $\delta V^{n}$, and gravity disturbance $\delta g^{n}$. And the velocity error $\delta V^{n}$ is the main error source that causes the position errors of INS.

Based on the above analysis, we can see that gravity disturbance $\delta g^{n}$ first leads to INS velocity errors in Equation (4), then further affects the position and attitude accuracy of INS through Equations (5) and (6). With the improvement of the accuracy of inertial sensors, INS solution errors due to gravity disturbance are becoming more and more significant and thus cannot be neglected; some measures must be taken to compensate for it.

**Figure 3:**
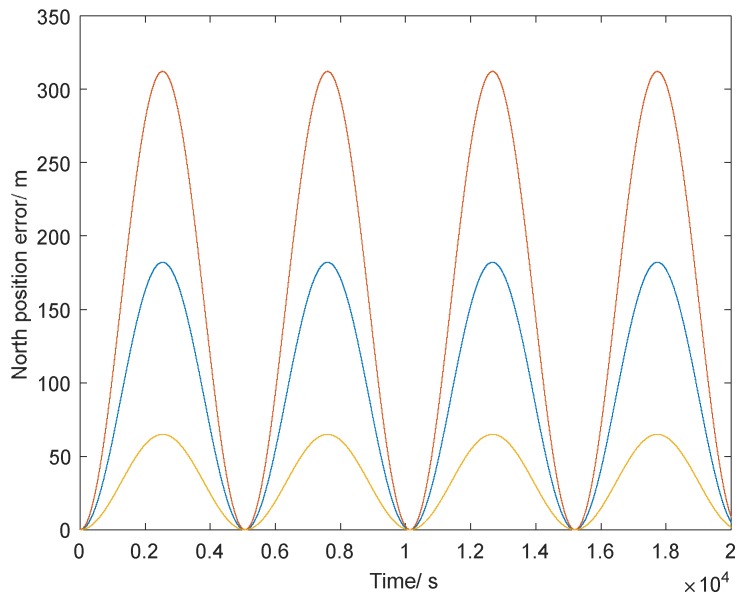
The north position error caused by gravity vertical deflection ζ.

Here we calculate the north position error caused by three typical values of north–south gravity vertical deflection ζ as an example to better represent the influence of gravity disturbance on INS. And the simulation results are shown in Table 1 and Figure 3.

Table 1 and Figure 3 show that the north position error of INS due to gravity vertical deflection propagates according to the Schuler cycle (about 84.4 min), and the amplitude of the position error is in direct proportion to the value of gravity vertical deflection. Thus, the influence of gravity disturbance in a high-precision INS cannot be neglected; it must be compensated to realize a more accurate navigation result.

.

## 3. The Principle of the Simplified Gravity Model

For a simplified reference, the Earth can be assumed to take the shape of an ellipsoid of revolution with a uniform mass distribution. There are some Earth models that can describe the Earth’s characteristics, such as WGS84, DQM2000, EGM2008, etc., which are normally used to describe the Earth’s gravitational field and are expressed with high degree spherical harmonic polynomials. Normally, in most inertial navigators, the WGS84 reference ellipsoid is used to calculate the normal gravity and the order of it only expands to 2 degrees. However, with the development of the gravity models, WGS84 cannot perfectly describe the gravitational field to meet the demands of high accurate inertial navigators. To further restrain the errors caused by the gravity model, the more accurate gravity model (EGM2008) is used to replace the WGS84 model to calculate the normal gravity. Although the EGM2008 model can well describe the characteristics of the Earth, it is hard to apply this gravity model with a high number of degrees to the solution of the INS because of the time and space complexity [7]. Thus, here a simplified gravity model is proposed to solve this problem.

### 3.1. Expression of Spherical Harmonic Gravity Model (SHM)

The gravitational potential of the earth to external point is a harmonic function, it can be represented by the infinite order number of a spherical harmonic function [20,21]:

(10) $$V_{\left(\rho ,\theta ,\lambda \right)}=\frac{fM}{\rho }[1-\sum_{n=2}^{\infty }{\left(\frac{a_{e}}{\rho }\right)}^{n}\sum_{m=0}^{n}\left({\bar{C}}_{nm}\cos(m\lambda )+{\bar{S}}_{nm}\sin(m\lambda ){\bar{P}}_{nm}(\cos\theta )\right)],$$

where (ρ,θ,λ) is the spherical coordinate with the center of the earth as the origin of the coordinate; f is denoted as the Newton’s gravitational constant; $a_{e}$ is the major radius of reference ellipsoid; M is the mass of the Earth; ${\bar{P}}_{nm}(\cos\theta )$ is associated Legendre polynomials, ${\bar{C}}_{nm},{\bar{S}}_{nm}$ are denoted as constant coefficients of the spherical harmonic of degree n and order m. Therefore, if these coefficient values are known, the value of the gravitational potentiation can be obtained. In order to get the value of the gravitational potentiation, the number of coefficients is limited. Thus, Equation (10) can be rewritten as follows:

(11) $$V_{\left(\rho ,\theta ,\lambda \right)}=\frac{fM}{\rho }[1-\sum_{n=2}^{N}{\left(\frac{a_{e}}{\rho }\right)}^{n}\sum_{m=0}^{n}\left({\bar{C}}_{nm}\cos(m\lambda )+{\bar{S}}_{nm}\sin(m\lambda ){\bar{P}}_{nm}(\cos\theta )\right)],$$

where *N* is the highest order of the known coefficients.

### 3.2. The Selection of the Degree for the Simplified Gravity Model

The covariance between the gravity potential and harmonic coefficients considers the degree of their correlation. The effect of certain coefficients on the gravity potential is reflected by a high value of the covariance. Therefore, we could theoretically use it to determine a suitable degree of SHM. In the gravity potential theory, Equation (11) can be changed into the following equation by multiplying both sides with a specific $P_{nm}(\cos\theta )\cos(m\lambda )$ or $P_{nm}(\cos\theta )\sin(m\lambda )$ and integrates over the unit sphere [22,23]:

(12) $$\begin{array}{c} C_{nm}\\ S_{nm} \end{array}\}=\frac{\rho }{4\pi fM}{\left(\frac{\rho }{a}\right)}^{n}\iint_{\sigma }VP_{nm}(\cos\theta )\left\{\begin{array}{c} \cos(m\lambda )\\ \sin(m\lambda ) \end{array}\right\}d\sigma ,$$

where dσ=sinθdθdλ is defined as the surface element of the unit sphere.

Defining $L^{C}$ and $L^{S}$ as a linear functional, so the Equation (12) can be simplified as follows:

(13) $$\begin{array}{c} C_{nm}\\ S_{nm} \end{array}\}=\left\{\begin{array}{c} L_{nm}^{C}\\ L_{nm}^{S} \end{array}\right\}V.$$

The linear functional is defined as the continuous analogue to the usual concept of a linear function in n-dimensional vector space. Therefore, the covariance between $C_{nm},S_{nm}$ and T satisfy the following condition:

(14) $$\mathrm{cov}(\left\{\begin{array}{c} C_{nm}\\ S_{nm} \end{array}\right\},V)=\left\{\begin{array}{c} L_{nm}^{C}\\ L_{nm}^{S} \end{array}\right\}K(P),$$

where K(P) is the covariance of the gravity potential on point *P*, described as

(15) $$\begin{array}{l} K(P)=\sum_{n=2}^{\infty }\frac{K_{n}}{2n+1}{\left(\frac{R}{\rho }\right)}^{2(n+1)}\\ \;\times \sum_{m=0}^{n}P_{nm}{\left(\cos\theta \right)}^{2}\cos(m\lambda ), \end{array}$$

where *R* is the Earth’s average radius, ρ is the geocentric radius distance of *P*, and $K_{n}$ is the degree variance of coefficients:

(16) $$K_{n}={\left(\frac{fM}{R}\right)}^{2}\sum_{m=2}^{n}\left(C_{nm}^{2}+S_{nm}^{2}\right).$$

Consequently, the covariance between the gravity potential and the harmonic coefficients can be described as

(17) $$\mathrm{cov}(\left\{\begin{array}{c} C_{nm}\\ S_{nm} \end{array}\right\},V)=\frac{K_{n}}{2n+1}\frac{R}{fM}{\left(\frac{R}{\rho }\right)}^{n+1}P_{nm}(\cos\theta )\left\{\begin{array}{c} \cos(m\lambda )\\ \sin(m\lambda ) \end{array}\right\}.$$

**Figure 4:**
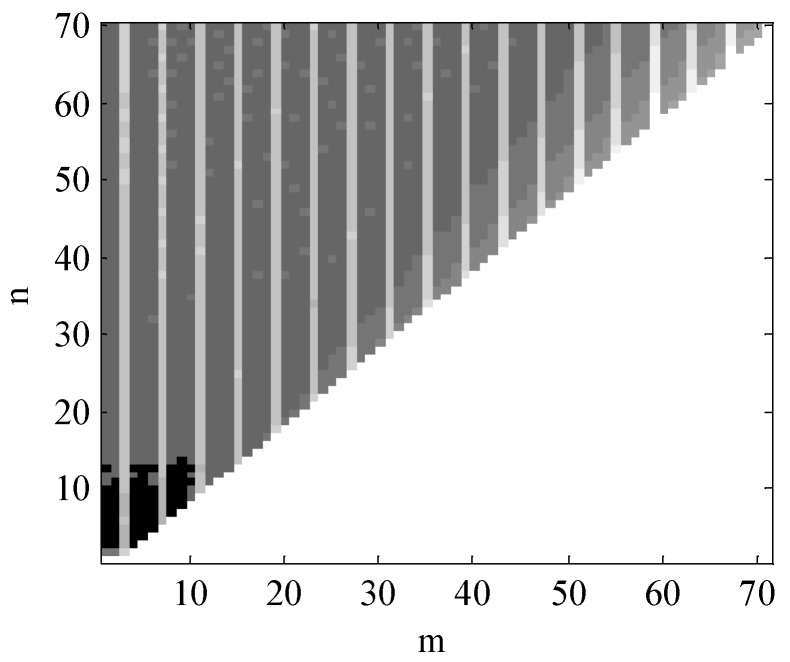
The common logarithm of $\mathrm{cov}{\left(C_{nm},V\right)}_{P}$ when $N_{\max}=70$.

**Figure 5:**
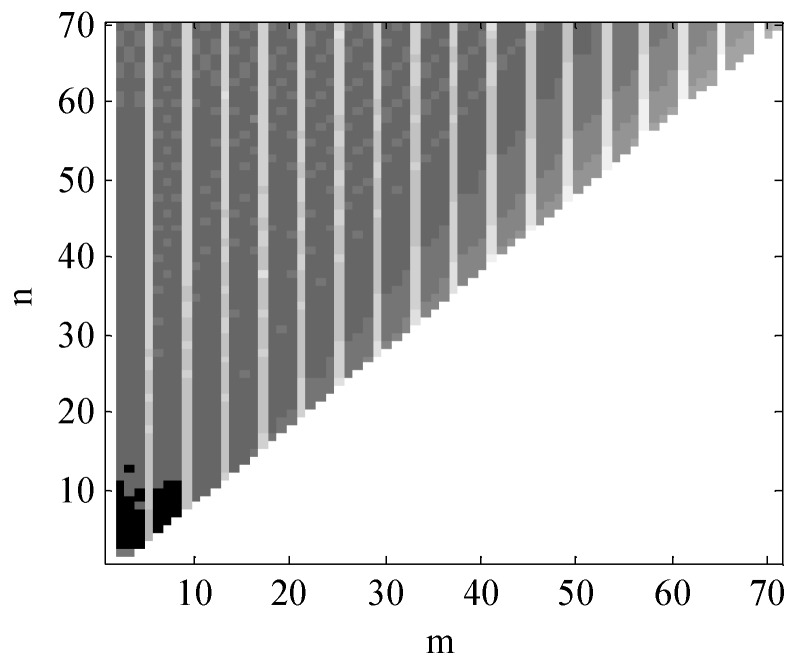
The common logarithm of $\left|\mathrm{cov}{\left(S_{nm},V\right)}_{P}\right|$ when $N_{\max}=70$.

The maximum number of degrees of EGM2008 is 2160 degrees, however, the high order terms have only little influence on the results of calculation. Thus, to better illustrate the number of orders of EGM2008 affecting on the results of calculation, here we set the maximum order ($N_{\max}=70$) to calculate the absolute magnitudes of $\mathrm{cov}{\left(C_{nm},V\right)}_{P}$ and $\mathrm{cov}{\left(S_{nm},V\right)}_{P}$. Since the covariance of the points around the world are similar, the point $P(\theta =45^{\circ },\lambda {=45}^{\circ })$ on the Earth’s surface is used for discussion. When $N_{\max}=70$, the absolute magnitudes of $\mathrm{cov}{\left(C_{nm},V\right)}_{P}$ and $\mathrm{cov}{\left(S_{nm},V\right)}_{P}$ range from $10^{-3}$ to $10^{-15}$ and $10^{-3}$ to $10^{-13}$, respectively. To better visualize the results, Figure 4 and Figure 5 are plotted with common logarithms of $\left|\mathrm{cov}{\left(C_{nm},V\right)}_{P}\right|$ and $\left|\mathrm{cov}{\left(S_{nm},V\right)}_{P}\right|$ respectively, shown as follows:

In Figure 4 and Figure 5, horizontal ordinate (m) and longitudinal coordinate (n) represent the number of degrees of the EGM2008. In these two figures, the darker place means the greater value of logarithms of $\left|\mathrm{cov}{\left(C_{nm},V\right)}_{P}\right|$ and $\left|\mathrm{cov}{\left(S_{nm},V\right)}_{P}\right|$. From these two figures, we could see that the values of the covariance are mainly focused below 12 degrees, which indicates the value of gravity mainly focuses on the SHM’s lower degree. Thus, in this paper, we set the maximum order of the simplified SHM as $N_{\max}=12$ to calculate the normal gravity.

## 4. The Principle and Procedure of the Data-Based Gravity Disturbance Compensation Method for INS Using ELM

### 4.1. The Principle of ELM

In recent years, ELM has been becoming a popular research method to solve many engineering problems. ELM has its own distinguishing features, which are depicted as follows [24]:

(1) Only the predefined network structure needs to be modulated;
(2) ELM has the ability to do fast learning;
(3) High generalization performance can be achieved through ELM;
(4) A wide selection range of activation functions can be used in ELM.

Due to its many advantages, in this paper, an estimation method based on ELM is used to estimate the gravity disturbance on the trajectory of the motion carrier, then compensate it into the INS solution equations to improve the navigation accuracy.

The principle of the ELM is described as follows [25]:

Given that we have a training set ${\left\{\left(x_{i},t_{i}\right)\right\}}_{i=1}^{N}$ with *N* distinct examples, where $x_{i}={\left[x_{i1},x_{i2},x_{i3},\ldots ,x_{in}\right]}^{T}$ has n inputs and $t_{i}={\left[t_{i1},t_{i2},t_{i3},\ldots ,t_{im}\right]}^{T}$ has m outputs. Here we define each $x_{i}$ has longitude and latitude two values, described as $x_{i}=[\lambda_{i}\;L_{i}]$ and the output $t_{i}$ is the gravity disturbance on geoid, described as $t_{i}=[\Delta g_{Ei}\;\Delta g_{Ni}\;\Delta g_{Ui}]$. Here l is denoted as the number of hidden neurons, ω is denoted as the l×n input weight matrix where $\omega_{j}={\left[\omega_{j1},\omega_{j2},\omega_{j3},\ldots ,\omega_{jn}\right]}^{T}$, b is the l×1 biases vector and β is the l×m output weight matrix where $\beta_{j}={\left[\beta_{j1},\beta_{j2},\beta_{j3},\ldots ,\beta_{jm}\right]}^{T}$. Generally, the ELM network function is shown as

(18) $$t_{i}=\sum_{j=1}^{l}\beta_{j}g\left(\omega_{j}\cdot x_{i}+b_{j}\right),\;i=1,2,\ldots ,N;$$

where $j\in \left\{1,2,\ldots ,l\right\},\;\omega_{j}\cdot x_{i}$ is defined as the inner product of $\omega_{j}$ and $x_{i}$, the activation function *g*(*x*) chooses sigmoid function in the proposed method, which is formulated as

(19) $$g\left(x\right)=\frac{1}{1+exp\left[-\left(\omega \cdot x+b\right)\right]}$$

Equation (18) can be simplified as the following simple form:

(20) Hβ=T

where

(21) $$H={\left[\begin{array}{ccc} g(\omega_{1}\cdot x_{1}+b_{1}) & \cdots & g(\omega_{l}\cdot x_{1}+b_{l})\\ \vdots & \ddots & \vdots \\ g(\omega_{1}\cdot x_{N}+b_{1}) & \cdots & g(\omega_{l}\cdot x_{N}+b_{l}) \end{array}\right]}_{N\times l}$$

*H* is denoted as the hidden layer output matrix of the network. *T* is the output matrix and $T={\left[t_{1},t_{2},\ldots ,t_{N}\right]}^{T}$.

According to ELM theory, the input weights and hidden biases can be selected at random, and the output weights can be obtained by calculating the following formula:

(22) $$\beta =H^{+}T$$

where *H*^+^ is the Moore-Penrose (MP) generalized inverse of matrix *H*.

### 4.2. The Procedure of the Data-Based Gravity Disturbance Compensation Method in INS Using ELM

The process of the data-based gravity disturbance compensation method for INS using ELM is described as follows:

1. Obtain the motion carrier’s position. Get the position information (*λ*, *L*) of the motion carrier through the INS calculation.
2. Choose the gravity data base. Find the suitable gravity data base taking the position obtained by step 1 as the center. Normally, 5′ × 5′ gravity grid data base is chosen as the training database.
3. ELM training. Set the motion carrier’s position information (*λ*, *L*) as the inputs of the network and acquire the gravity disturbance on geoid ($\Delta g_{E0},\Delta g_{N0},\Delta g_{U0}$) through training the gravity data base acquired by step 2.
4. Upward continuation. Calculate the gravity disturbance with upward continuation to the point where INS is. The height of INS is acquired through altimeter. In the geographic engineering application, the most practical upward continuation method is free air correction. The computational equation is described as follows [26]:
  (23) $$\Delta g=\Delta g_{0}-0.3086H$$
  where Δg is the gravity disturbance where the motion carrier is and $\Delta g_{0}$ is the gravity disturbance on the geoid. The units of Δg and $\Delta g_{0}$ are milligal. *H* is the height between the geoid and the motion carrier. The unit of *H* is meter.

## 5. The Framework of the Combined Gravity Compensation Method for INS

**Figure 6:**
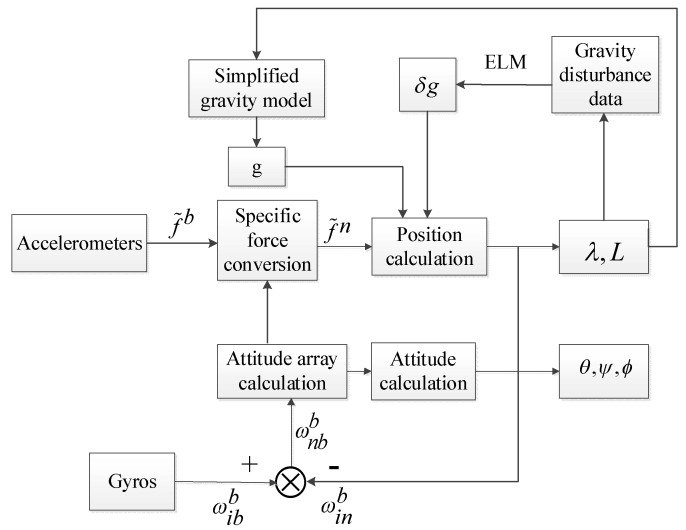
The flow chart of the combined gravity compensation method in the inertial navigation system (INS).

The flow chart of the combined gravity compensation method for INS is illustrated in Figure 6:

The process of the combined gravity compensation method is described as follows:

1. Obtain the motion carrier’s position. Get the position information of the motion carrier through the INS calculation.
2. Calculate the gravity and the gravity disturbance. Based on the position information from step 1, the gravity *g* and the gravity disturbance δg are obtained respectively through the simplified gravity model and ELM training.
3. Compensate the gravity and the gravity disturbance from step 2 into INS equations, considering the gravity disturbance to restrain the error propagation.

## 6. Experiment

**Figure 7:**
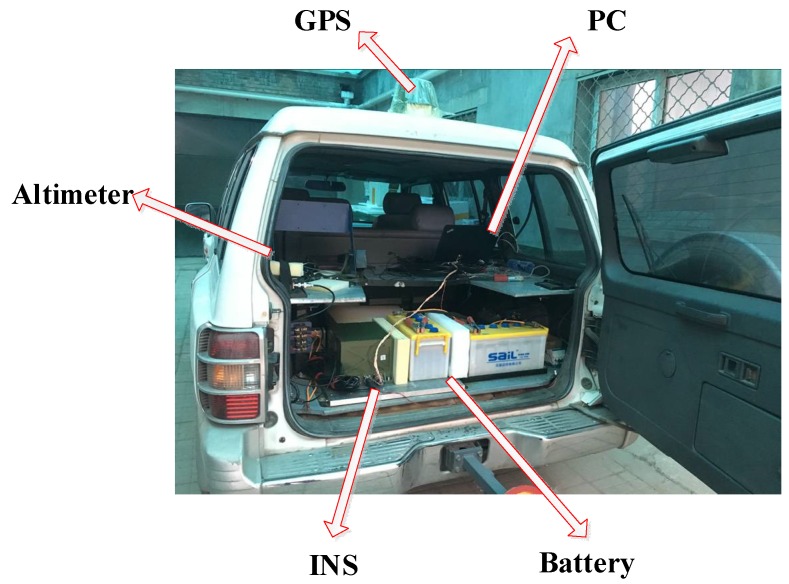
Field test device.

To prove the effectiveness and feasibility of the combined gravity compensation method in INS, two vehicle tests were applied in the city of Xi’an in China and denoted as test 1 and test 2. Test 1 and test 2 were applied in the areas with flat terrain and complex terrain respectively. On the test vehicle, an altimeter, a GPS receiver, a set of batteries, a high-precision INS, and a data processing computer (PC) were carried. The high-precision INS and the batteries were fixed on the overplate at the trunk. The computer was used to collect the data of GPS and INS in real time, then the data was resolved for navigation. The test vehicle is shown in Figure 7. The accuracy of the inertial sensors and GPS are listed in Table 2.

**Figure 8:**
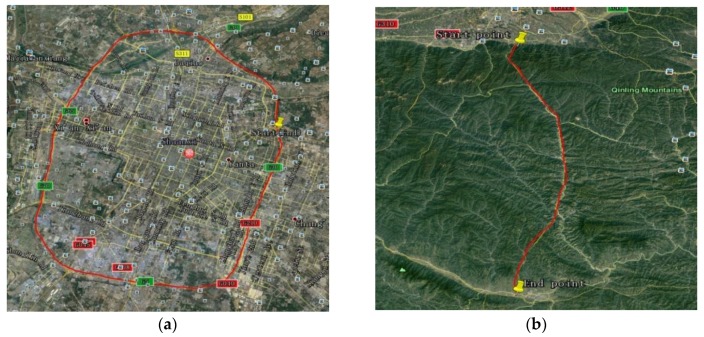
Test profiles on Google map. (**a**) Test 1; (**b**) Test 2.

**Figure 9:**
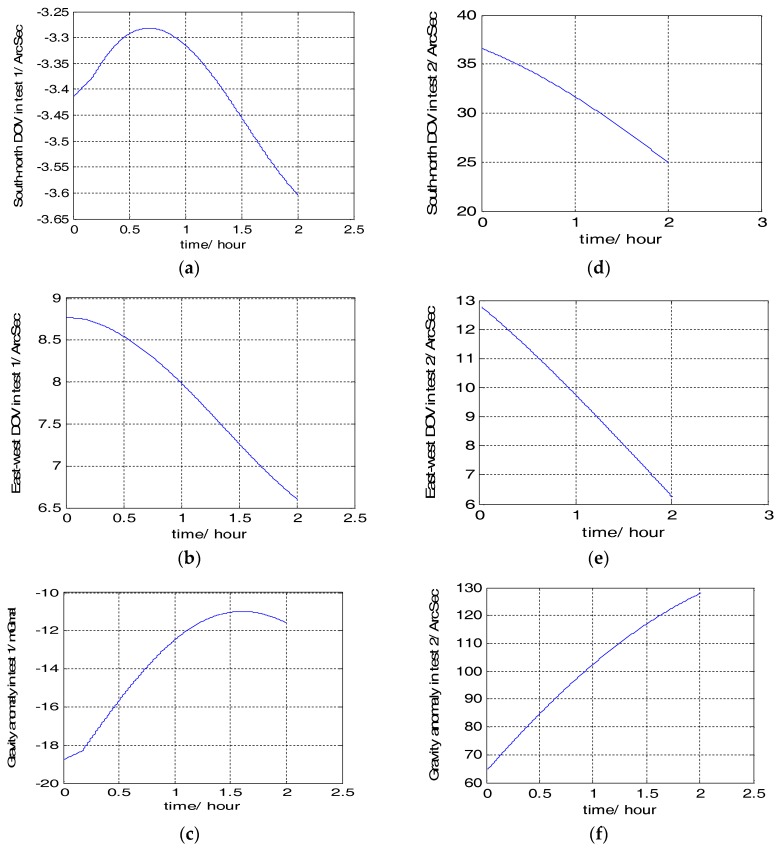
The gravity anomaly and DOVs of two tests. (**a**) South-north DOV in test 1; (**b**) East-west DOV in test 1; (**c**) Gravity anomaly in test 1; (**d**) South-north DOV in test 2; (**e**) East-west DOV in test 2; (**f**) Gravity anomaly in test 2.

The travel profiles, gravity anomalies and DOVs of the two tests are illustrated in Figure 8 and Figure 9. In Figure 9, the gravity anomalies and DOVs of the two tests were obtained from the estimation based on the measured gravity data using ELM training. The gravity data is provided by the Institute of Geodesy and Geophysics; Chinese Academy of sciences.

**Figure 10:**
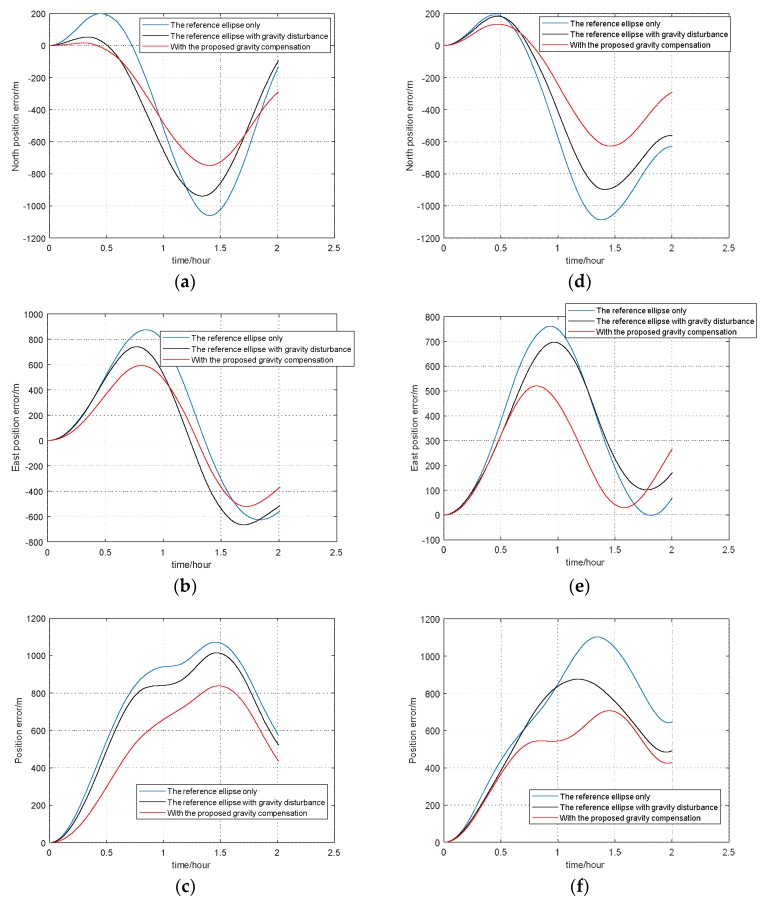
Position errors of two tests. (**a**) North position error of test 1; (**b**) East position error of test 1; (**c**) Position error of test 1; (**d**) North position error of test 2; (**e**) East position error of test 2; (**f**) Position error of test 2.

From Figure 9, we can see that the values of DOVs and gravity anomaly in the area of test 1 are both larger than that of test 2. To better prove the effectiveness and accuracy of the combined gravity compensation method, the maximum position errors were used to prove this. Here we regarded the position result of GPS as the true value, and respectively compared it with the position results compensated with three different gravity compensation methods: 1 only with the reference ellipse (shown as blue colour); 2 with the reference ellipse and gravity disturbance (shown as black colour); 3 with the proposed compensation method (shown as red colour).The position error results are shown in Figure 10, in which (a), (b), (c) are the position results of test 1; (d), (e), (f) are the position results of test 2.

According to Figure 10, the maximum position errors of INS compensated with three different methods in two tests are listed in Table 3.

From Figure 10 and Table 3, we could draw the conclusion that the proposed gravity compensation method performed well in two tests, especially in test 2 with a wider variation range of gravity disturbance. During the 2 h vehicle tests, compared with the solution results compensated with the reference ellipse only, the maximum value of position errors can reduce by 20% and 38% respectively, in test 1 and test 2, with the proposed gravity compensation method. Thus, the effectiveness of this combined gravity compensation method for INS is proved.

## 7. Conclusions

This paper proposed a combined gravity compensation method for high-precision INS. The method utilizes the simplified gravity model to subtract the normal gravity from the navigation equations, and meanwhile compensates the gravity disturbance through the ELM training based on measured gravity data. The effectiveness and accuracy of the combined gravity compensation method were verified by vehicle tests, which were applied with a high-precision INS, and the solution results show that the maximum position errors can reduce by 20% and 38%, respectively, in flat terrain and complex terrain. It should be noted that in this work the tests were carried on an experimental vehicle running on the surface of the Earth with relatively low speed. Therefore, in the follow-up study, a flight test with a relatively high travel speed should applied to further test the applicability of this gravity compensation method.

## Figures and Tables

**Table 1:** The simulation results of north position error caused by typical gravity vertical deflection.

	ζ=1sec	ζ=3sec	ζ=5sec
$\Delta g_{N}$ (m Gal *)	5	14	24
North position error (m)	62	185	309

* 1 m Gal = 1 × 10^−5^ m/s^2^.

**Table 2:** Accuracy of the sensors for the test.

Sensors Types	Characteristics	Magnitude (1 *σ*)
Gyroscope	Constant Bias	0.003°/h
Accelerometer	Constant Bias	10 μg
GPS velocity	Horizontal error	0.03 m/s
	Height error	0.05 m/s
GPS position	Horizontal error	2 m
	Height error	5 m

**Table 3:** The maximum value of position error compared with GPS result (Unit: meter).

	The Reference Ellipse Only	The Reference Ellipse with DOVs	With the Proposed Gravity Compensation	Position Improvement (Compared with the Reference Ellipse Only)
Test 1	1050	1012	837	213(20%)
Test 2	1120	876	689	431(38%)
